# Socio-Economic Variation in Price Minimizing Behaviors: Findings from the International Tobacco Control (ITC) Four Country Survey

**DOI:** 10.3390/ijerph8010234

**Published:** 2011-01-20

**Authors:** Andrea S. Licht, Andrew J. Hyland, Richard J. O’Connor, Frank J. Chaloupka, Ron Borland, Geoffrey T. Fong, Nigar Nargis, K. Michael Cummings

**Affiliations:** 1 Department of Health Behavior, Roswell Park Cancer Institute, Elm and Carlton Streets, Buffalo, NY 14263, USA; 2 Department of Social and Preventive Medicine, School of Public Health and Health Professions, University at Buffalo, Buffalo, NY 14214, USA; 3 Department of Economics, University of Illinois at Chicago, Chicago, IL 60607, USA; 4 Vic Health Center for Tobacco Control, The Cancer Council Victoria, Carlton, VIC 3053, Australia; 5 Department of Psychology, University of Waterloo, Waterloo, Ontario N2L 3G1, Canada; 6 Ontario Institute for Cancer Research, Toronto, Ontario M5G 0A3, Canada

**Keywords:** tobacco, policy, price, tax, socio-economic status

## Abstract

This paper examines how socio-economic status (SES) modifies how smokers adjust to changes in the price of tobacco products through utilization of multiple price minimizing techniques. Data come from the International Tobacco Control Policy Evaluation (ITC) Four Country Survey, nationally representative samples of adult smokers and includes respondents from Canada, the United States, the United Kingdom and Australia. Cross-sectional analyses were completed among 8,243 respondents (7,038 current smokers) from the survey wave conducted between October 2006 and February 2007. Analyses examined predictors of purchasing from low/untaxed sources, using discount cigarettes or roll-your-own (RYO) tobacco, purchasing cigarettes in cartons, and engaging in high levels of price and tax avoidance at last purchase. All analyses tested for interactions with SES and were weighted to account for changing and under-represented demographics. Relatively high levels of price and tax avoidance behaviors were present; 8% reported buying from low or untaxed source; 36% used discount or generic brands, 13.5% used RYO tobacco, 29% reported purchasing cartons, and 63% reported using at least one of these high price avoidance behaviors. Respondents categorized as having low SES were approximately 26% less likely to report using low or untaxed sources and 43% less likely to purchase tobacco by the carton. However, respondents with low SES were 85% more likely to report using discount brands/RYO compared to participants with higher SES. Overall, lower SES smokers were 25% more likely to engage in at least one or more tax avoidance behaviors compared to their higher SES counterparts. Price and tax avoidance behaviors are relatively common among smokers of all SES strata, but strategies differed with higher SES groups more likely to report traveling to a low-tax location to avoid paying higher prices, purchase duty free tobacco, and purchase by cartons instead of packs all of which were less commonly reported by low SES smokers. Because of the strategies lower SES respondents are more likely to use, reducing price differentials between discount and premium brands may have a greater impact on them, potentially increasing the likelihood of quitting.

## 1. Introduction

Large disparities are present in use of tobacco products across socio-economic strata, with higher smoking prevalence observed among persons with lower SES. Findings from a U.S. Center for Disease Control and Prevention (CDC) report indicate that the socioeconomic status of U.S. adults is inversely related to their likelihood of smoking [1]. Smoking prevalence is also highest among adults who have only earned a GED and is lowest among people with graduate degrees [1–3]. Additionally, the prevalence of smoking is also higher among those living below the poverty line [1].

Raising cigarette prices has been shown to be an effective tobacco control policy [4–9] and results in decreased cigarette consumption, increased quit attempts, and higher rates of smoking cessation [7,8,10–12]. Econometric evidence has suggested that price increases are more effective in reducing smoking among adults with lower income [2,3,12–14] than those with higher incomes. Thus, increasing the monetary cost of tobacco, particularly through taxation, may present major opportunities to reach more deprived smokers as they will be less able to afford their usual smoking pattern after a price increase [12]. Cigarette excise taxes have been typically viewed as regressive, in which the tax burden is relatively greater among those with lower rather than higher incomes. However, among those who quit, the poor can incur a greater benefit from tax increases by gaining both extra money and improved health [15]. In general, public health advocates usually support higher cigarette taxes because of the health harms caused by smoking and its demonstrated capacity to reduce consumption.

Based on a compensatory model developed by ITC investigators [16], smokers faced with price or tax increases may engage in a number of behaviors to reduce the overall impact of the price increase including quitting or cutting back on cigarettes smoked. Less desirable behaviors may include finding cheaper sources of tobacco, either by buying from legitimate discount outlets, buying in bulk (e.g., in cartons) or though avoiding taxes or purchasing from reduced tax sources. Additionally, smokers may switch to cheaper forms of tobacco. It is well established that price increases reduce consumption by a mixture of increased cessation and cutting down [5–8,11,17,18]. However, much less information is available on similar estimates of price elasticity for these cost avoidance compensatory behaviors. This manuscript focuses on these behaviors which smokers can engage in to help alleviate the price burden and assesses use of these alternative behaviors across various socio-economic strata.

### Price Reduction Behaviors

Smokers may change their usual purchasing behaviors in response to a price increase. Following a large tax increase affecting cigarettes purchased within New York City limits, the proportion of cigarettes reportedly purchased outside of the city limits increased by 89%. Among respondents who reported purchasing cigarettes elsewhere, most alternative purchases were from lower taxed jurisdictions such as purchasing cigarettes in New York State (outside of the city), in a different state, over the internet, from another person, or from an Indian Reservation [7]. Additionally, after this same tax increase, many low income and minority smokers also admitted to purchasing cigarettes from a widening network of independent bootleggers, dubbed the “$5 men” [19]. Smokers may also make purchases from other sources that would result in a lower overall price, including Indian Reservations in North America [10,11,20,21] or outside the state/country of their residence [10,11,22].

From a 2005 study of US smokers, less than 30% of smokers overall indicated that price was a motivating factor in choosing the brand they smoked; however lower income smokers were more likely to smoke a cheaper brand after adjustment in multivariate modeling [23]. Other literature suggests that smokers may switch to different tobacco types, especially from manufactured to self-made or RYO cigarettes [24,25], or choosing brands that have higher nicotine yield [26].

Based on previous literature, persons who engage in tax avoidance behaviors by purchasing from untaxed sources such as Indian Reservations, the internet, or from independent sellers, are more likely to be: female [10,20,27], older [10,11,20,23], have higher incomes [10,11,23], live closer to sources of less expensive cigarettes [10,20,22], have higher daily cigarette consumption [10,11,21,23,27], and have no plans to quit smoking [21]. Conversely, persons with lower incomes are more likely to smoke discount or generic brand cigarettes [10,23,28]. However smokers of discount or generic cigarettes overall are more likely to smoke a greater number of cigarettes per day [10,23,28], and smoke their first cigarette within a shorter time of waking [10]. The literature has also shown that African Americans [10,28] and Hispanics [28] are less likely to smoke discount or generic cigarettes compared to whites.

This paper presents results of an analysis examining the extent to which smokers in four different countries—the United States, Canada, Australia, and the United Kingdom—engage in behaviors to minimize the impact of higher cigarette prices and how these behaviors differ among varying socio-economic groups. Past literature has shown given some insight into the issue of price avoidance techniques and has described who is using them [7,10,11,19–21,23,27,28]. However, such literature has been limited, in that many describe use in a specific population [19,20] or state/province [21,23,27] and describe the effects of a single event such as a tax increase [7,19,20]. The current study reports a comprehensive description of multiple price and tax avoidance techniques in an international population of smokers. Additionally, this study also assesses differential use of these strategies by varying SES groups. SES has been shown to be a strong predictor of smoking status and there is evidence of a widening gap in the social inequalities of smoking prevalence. The data presented in this paper assess the prevalence of purchasing discount cigarettes or RYO tobacco, utilizing low and untaxed sources, or purchasing cigarettes in cartons by country; demographic and behavioral predictors of seeking out lower priced cigarettes; and the impact of socio-economic status (SES) on price minimizing behaviors.

## 2. Experimental Section

### 2.1. Data Source

Data came from the International Tobacco Control (ITC) Four Country Survey (ITC-4 Survey), whose conceptual framework and methodology are described in greater detail in earlier publications [29,30]. The ITC-4 Survey aims to evaluate psychosocial and behavioral outcomes following from the implementation of provisions of the WHO Framework Convention on Tobacco Control (FCTC) [29]. It follows a “quasi-experimental” design, employing both cross-sectional and longitudinal study arms, to allow researchers to make strong inferences about policy effects [29]. The ITC-4 Survey recruits adult smokers in the United States (US), Canada (CA), the United Kingdom (UK), and Australia (AU), but follows them up regardless of subsequent quitting. At least 2,000 participants from each country are interviewed at each survey wave; however, due to attrition, the sample is replenished at each survey wave to maintain adequate sample. All ITC participants are identified using stratified random digit dialing regardless of whether they were part of the continuing cohort or are newly recruited to compensate for loss to follow-up. Therefore, any given replenishment sample is representative of the population at the time of data collection rather than those lost to follow-up [30]. All interviews were conducted using computer assisted telephone interview (CATI) software by multiple research facilities, with strict protocols in place to ensure methodological consistency [29,30].

### 2.2. Study Population

The present study conducted analyses on cross-sectional data collected between October 2006 and February 2007. Completed interviews were obtained from 8,243 respondents, of which 7,038 (85%) were current smokers; 1,741 in Canada, 1,790 in the US, 1,706 in the UK, and 1,801 in Australia. Among these completed interviews, 2,638 participants were newly recruited at this survey wave (2006–2007) to replace cohort members who were lost to follow-up from the previous survey wave. Loss to follow-up was essentially the same across the four countries.

### 2.3. Other Covariate Measures

Covariates included in all multivariate analyses were country of residence (Canada, the United States, the United Kingdom, and Australia); age at time of interview (18–39, 40–54, 55+); sex (female, male); minority status (identified minority *vs.* all others. In CA, US and UK race was used (white *vs.* non-white), in Australia it was defined based on speaking English at home (yes, no)); and cigarettes per day at time of interview (≤10, 11–20, 21–30, ≥31).

### 2.4. SES Composite Variable

The main focus of this manuscript is to assess whether a respondent’s socio-economic status (SES) moderated the relationship between other predictor variables and use of specific purchasing behaviors.

Education level was assessed by asking “What is the highest level of formal education that you have completed?” Responses varied by country due to different education systems, but were recoded into three comparable categories: (1) low education (completed high school or less); (2) moderate education (training, technical school, some university); or (3) high education (university degree or higher).

Average annual household income was derived from a question asking respondents about their annual household income. Responses varied depending on country of residence to account for different monetary systems and were categorized into three levels, after treating £1 UK as equivalent to $2 USD. The dollar amounts of the other three other countries are treated as equivalent. Categories were: (1) low income (≤$30,000/≤£15,000); (2) moderate income ($30,000–59,000/£15,000–30,000); or (3) high income (≥$60,000/≥£30,000). Those who did not provide their annual income were excluded from the analyses.

The SES composite measure combined income and education into a low, moderate, high scale. Participants with low education and low income were categorized as having “low” SES. Those with any combination of moderate or high education and income were deemed to have “high” SES. All other combinations were categorized as being “moderate” SES. Sensitivity analyses that combined income and education into different SES strata did not produce varying results. The SES distribution of the smokers (n = 7,038) included 18.8% with low SES, 40.3% with moderate SES, and 33.8% with high SES. SES could not be assessed in 7.0% of smokers in the main study population due to missing data, mainly due to missing income.

### 2.5. Definition of Outcome Measures

Purchasing discount/generic or RYO tobacco was defined based on the self-reported brand and variety last purchased and was used as a proxy for recent brand/variety exposure. All varieties of a given brand were treated equivalently (e.g., full flavor, light, or menthol) and were categorized as being premium brands, discount brands, or “roll your own” (RYO) varieties based on previous work [31] and current cigarette market research. Table 2 reports all specific brands reported and whether they were coded as being premium, discount, or RYO tobacco, by country. Table 3 shows the frequency of premium, discount, or RYO tobacco. For analyses predicting use of these behaviors (Table 4), RYO and discount/generic brands users were combined because similar results were obtained when only discount users were included in the analysis.

Assessment as to whether the last purchase was from a low or untaxed venue was based on the reported last purchase location. Response categories included (1) Convenience store, gas station, newsstand; (2) Grocery store, discount/”big box” outlet stores; (3) Discount tobacco outlet venues or tobacco specialty shops; (4) entertainment venues such as bars, restaurants, casinos; (5) liquor stores; (6) from a vending machine; (7) Military commissaries; (8) Duty-free shops; (9) Indian Reservations; (10) Outside the state/country of residence (11) by Internet or telephone; or (12) from a friend, relative, or other independent seller. ‘Low/untaxed purchasers’ were taken as those from: military commissaries (US only), Indian Reservations (US and CA only), duty free shop, outside the state or country, by telephone, the internet, someone else, or a friend or relative, with all other sources treated as full taxed venues.

Current smokers who reported purchasing factory made cigarettes at last purchase were queried on the unit of tobacco last purchased (carton, pack, or loose/single cigarettes). Respondents who purchased tobacco in a carton at last purchase were considered to be participating in a price minimizing behavior while those who reported purchasing packs or single/loose cigarettes were not. Respondents who purchased RYO tobacco at last purchase were excluded from this construct.

A composite construct to assess any use of avoidance/minimization strategies was also computed. Respondents were given a score of “1” for each of the preceding price minimizing behaviors for which they reported using. Individual scores were added to obtain a measure of any price and tax avoidance at last purchase. For smokers of factory-made cigarettes, respondents could obtain a maximum score = 3 if they used a discount brand purchased in a carton from a low or untaxed source. For RYO tobacco users, a maximum score = 2 could be obtained, which included RYO tobacco users who purchased from low or untaxed sources. This price and tax avoidance score was categorized into “no use” (score = 0) *vs.* “any use” (score ≥ 1) at last purchase.

Price paid per cigarette was computed for last purchase using price they paid per unit of tobacco (carton, pack, loose) or the total price paid for the last tobacco purchase, the number of cartons/packs, and the number of cigarettes per pack. Reported prices per cigarette were adjusted for currency and inflation, and are all reported in US dollars for the year 2006. Outliers, defined as values outside of 3 standard deviations of the mean, were excluded. RYO users were also excluded from this analysis, as a ‘price per cigarette’ could not be calculated.

### 2.6. Statistical Methods

All analyses were weighted to adjust for deviations in the age and sex distribution of the sample compared to the population and for replenishment into the cohort [30]. Weighting techniques and procedures are published elsewhere [30]. All analyses were completed using SPSS version 14.0. Univariate analyses were used to describe the study population and the frequency at which respondents reported each price minimizing behavior. Analyses were stratified by country and differences were assessed using the chi square test for independence. Multivariate logistic regression modeling was used to assess demographic and behavioral predictors of purchasing from low/untaxed sources, using discount brands, purchasing tobacco in cartons, and use of any price or tax avoidance behaviors. Multiplicative interaction terms for each predictor variable and the SES composite variable were entered into multivariate logistic regression models to assess the joint effects of each on use of tax or price avoidance behaviors. Stratified analyses by SES were performed when a statistically significant interaction was present to assess the likelihood of utilizing the price or tax avoidance behaviors above in each population sub-group. P-values < 0.05 were considered to be statistically significant.

## 3. Results

Demographic characteristics of study participants included in this manuscript are presented in Table 1. Characteristics are given for the entire sample, and are also stratified by country. The frequency of reporting price minimizing strategies at last purchase is given in Table 3, stratified by country. Approximately 36% of all participants reported using discount or generic brand cigarettes. Use of discount/generic brands was highest among UK residents and lowest in the US. Overall, 13.5% of respondents used RYO tobacco at last purchase. In stratified analysis by country use of RYO tobacco was highest in the UK (29%), and lowest in the US (2.6%). Slightly less than a third of all respondents reported their last purchase was in carton-form. By country, purchasing by carton was highest in the US (41.2%) and lowest in Australia (17.2%) (p < 0.001). Combining these behaviors into a measure of any price and tax avoidance, 63% of respondents participated in at least one price or tax avoidance behavior at last purchase.

Table 4 presents demographic and other predictors of various price minimizing behaviors at last purchase. Regression analyses revealed that respondents with low SES were approximately 26% less likely to utilize low and untaxed sources at last purchase compared to those with high SES (OR = 0.74, Table 4). Increasing age was associated with increased likelihood of use (p for trend < 0.001). Respondents aged 55 years or higher were more than twice as likely to use low and untaxed sources compared to those less than 40 years old. Moreover, utilization of low and untaxed sources was more likely among respondents who smoked more than 10 cigarettes per day; however, this association was not statistically significant for the heaviest smokers (greater than 30 CPD). No statistically significant interactions were present between SES and any covariate for utilization of low and untaxed sources overall.

Respondents with moderate (OR = 1.36, 95% CI: 1.21–1.85) and low SES (OR = 1.85, 95% CI: 1.59–2.15) were significantly more likely to report smoking discount/generic brands or RYO tobacco compared to participants with high SES (Table 4). Other characteristics which were significantly associated with an increased likelihood of discount or RYO use included being from the UK, increasing age, and increasing cigarettes per day. Overall, minority group members were less likely to report using discounts or RYO tobacco compared to non-minority respondents but an interaction was present between SES and minority group status on use of discount/generic or RYO tobacco (p = 0.027). After stratification by SES, in the low SES strata minority group members were approximately 50% less likely to use discounts or RYO tobacco products compared to non-minority group members (OR = 0.49, 95% CI: 0.34–0.70). However, minority group members in both the moderate (OR = 0.85, 95% CI: 0.66–1.09) and high (OR = 0.76, 95% CI: 0.58–1.00) SES strata were no more or less likely to use discount brands or RYO tobacco products compared to non-minority respondents.

Among factory-made cigarette users, respondents with low SES were less likely to purchase cigarettes in a carton (OR = 0.57, Table 4). Additionally, minority group members were about 30% less likely to purchase cartons. Contrary to the other two price and tax avoidance behaviors studied, respondents from the US were significantly more likely to report purchasing cigarettes in cartons. Increasing cigarettes per day and increasing age were strongly associated with purchasing tobacco in a carton at last purchase (Table 4). There were no statistically significant interactions between SES and other covariates on purchasing cartons at last purchase.

Lower SES was significantly associated with use of at least one price or tax avoidance behavior at last purchase (p for trend = 0.009). Compared to respondents with high SES, those with both moderate (OR = 1.15) and low SES (OR = 1.25) were more likely to engage in any price or tax avoidance behaviors at last purchase (Table 4). Again, respondents from the US and AU were significantly less likely than Canadian respondents to utilize price and tax avoidance behaviors, while respondents in the UK were over 2 times more likely than Canadian respondents to engage in these behaviors. Overall, both males and minority groups were over 30% less likely to utilize price avoidance techniques, and dose-responses effects were seen among varying age groups and CPD categories.

There was a statistically significant interaction between SES and minority status on using at least one price or tax avoidance behavior at last purchase (p = 0.016). Although low SES respondents were more likely to use any price/tax avoidance behaviors, this did not hold true for minority group members who had low and moderate SES. In stratified analysis including only low SES respondents, minority group members were approximately 55% less likely to use any strategy at last purchase (OR = 0.45, 95% CI: 0.31–0.64) compared to non-minority low SES respondents. Similarly, in stratified analyses of only moderate SES respondents, minority group members were about 30% less likely to use any price/tax avoidance (OR = 0.71, 95% CI: 0.56–0.90) compared to non-minority moderate SES individuals. Among high SES smokers, minority and non-minority smokers did not differ in “any use” of price/tax avoidance behaviors.

Large variation in the computed measure of price per cigarette was observed within countries and among price and tax avoidance behaviors at last purchase (Table 5). All price per cigarette measures are given as mean (±standard deviation) and have been adjusted to US currency ($USD) for the year 2006. Stratified by country, US respondents reportedly paid the least amount per cigarette ($0.18 ± 0.068), while respondents in the UK reported paying the highest price per cigarette ($0.43 ± 0.105). Compared to respondents who did not engage in price minimizing behaviors, respondents who reported using these strategies reported paying lower prices per cigarette. Purchases made from low and untaxed sources resulted in the largest price differential overall. Among all respondents, there was a 42% difference in the reported price of cigarette purchased and not purchased in a carton. Only a small difference in the reported price per cigarette was observed between premium and discount brands which may be attributed to high discount prices and utilization among UK smokers. Therefore, the discount/generic brand prices in the UK were disproportionately weighing the overall average. In analyses excluding UK smokers, there was a 16% difference in self-reported price per cigarette between premium and discount brands. Use of at least one of the preceding price/tax avoidance behaviors resulted in a lower mean price per cigarette overall, compared to no use of price or tax avoidance.

## 4. Discussion and Conclusions

These findings indicate that a sizeable percentage of international smokers engage in behaviors aimed at obtaining lower priced cigarettes, by purchasing cheaper tobacco brands, utilizing low or untaxed tobacco retail sources, using self-made (RYO) tobacco products, or purchasing tobacco in bulk (carton purchases). Utilization of these price and tax avoidance behaviors may decrease the public health benefits of increasing cigarette prices through taxation by reducing the amount of quitting, especially among more deprived sub-populations.

Overall, low SES smokers were about 25% more likely to utilize at least one price or tax avoidance strategy at last purchase and were far more likely to use two of the more common price-reduction strategies of RYO tobacco and using discount brands. Strategies used less often by low SES respondents mainly involved some additional up-front cost, either in travel to a low-tax place, international travel, or in buying cartons. This is consistent with previous literature from the United States, where purchases by the carton are most frequent [23], and is to be expected as poorer people typically have less liquid resources.

These results are consistent with the argument that reducing price differentials between various tobacco alternatives may serve to reduce tobacco use disparities between SES groups, as low SES smokers would have less room to move. One way to accomplish this is having uniformly high tobacco prices across all jurisdictions and tobacco products, though current approaches have failed to do so. Minimum pricing policies may be able to accomplish this; however such laws must be comprehensively defined as to deter the tobacco industry from using other price promotion strategies that can be targeted to particular brands. Such tobacco industry strategies can artificially lower the price of cigarettes below the minimum price level, and therefore have little or no effect on the actual price [32].

Additionally, replacing state or provincial excise taxes with higher federal specific excise taxes may be another method to reduce price differentials where states get a portion of the tax revenue proportionate to consumption in the state. Specific taxes are based on quantity, not price, and may help minimize price gaps between premium and discount brands [33]. Mixed tax structures apply higher tax rates to higher priced brands, and may actually increase the price gap between discount and premium brands. Thus, mixed tax structures may create an incentive to switch to discount brands in response to a price increase. On the other hand, with specific taxes, a given tax increase would reduce the price gap between premium and discount brands, increasing the price ratio and thus potentially reducing the probability of smoking a discount brand [33]. However, with this approach, it should be done under the provision that the state has a comprehensive tobacco control program in place, or had to reduce its existing taxes as part of the harmonization process.

Moreover, as many low SES smokers are already using cheaper brands, the change would almost certainly differentially increase the cost of the cheaper brands, which would provide stronger incentives for poorer smokers to quit. However, it would also cause hardship among those unable to quit because they would be likely to spend more of their limited income on tobacco.

Previous data has suggested that use of low and untaxed sources may be dependent on a combination of high tobacco prices and relatively high availability of untaxed tobacco sources [11]. The results of this study confirm that use of low/tax avoidance strategies is largely driven by opportunity. These price-avoidance strategies were most common in the UK, where international travel is most frequent, leading to greater accessibility of low or untaxed tobacco products [11,34] and is least likely in Australia, probably due to the country’s relative isolation [11,35]. However, our results give mixed support for the role of price in tax avoidance purchases. The UK has had high cigarette taxes, and thus high prices [11,35]. However, Australia also has high prices, but has the lowest use of low and untaxed tobacco products among the four countries. This suggests that ease of tax evasion is a more critical factor.

A 2002 cross-sectional survey of adult smokers in California, USA, found that nearly 75% of respondents reported using at least one price minimizing strategy [23], a prevalence that is slightly higher than that found in the current study. However, the California study used a broader classification of price-minimizing behaviors, including use of promotional offers and purchasing from a cheaper outlet, which may partially explain the higher prevalence. In comparing individual sources studied, frequency of use of both low/untaxed sources and cheaper brands were generally comparable. However, the reported frequency of use of carton purchases was much higher in our current study compared to the previous cross-sectional study from 2002. One possible explanation for this difference could be due to increased state excise taxes on tobacco products between the time periods. This could have lead smokers to look for alternative, legal behaviors to reduce the cost of cigarettes, of which purchasing by the carton may be a viable option.

Our study found low levels of use of low/untaxed sources of tobacco that can be accessed readily: internet and other mail-order sales were low, and there was not a lot of evidence of street marketing of smuggled or other tax-unpaid tobacco. Other studies have noted that these types of cigarette sales, such as internet or mail-order sales may be under-reported due to issues of legality or due to the inconveniences associated with long wait times or minimum purchase requirements [36]. As noted above, this may be conflated with RYO use as illicit RYO tobacco is more widely available than illicit factory-made cigarettes, at least in the UK and Australia [37]. Due to Australia’s relative isolation, illicit trade of factory-made or contraband tobacco products remains a small share of the total tobacco market, although sales appear to be slowly increasing there [37].

Previous literature from the United States and abroad have shown that the persons living closer to low or untaxed sources are more likely to participate in tax evasion behaviors [10,20,22]. These findings suggest that some resources may be necessary in order to utilize low and untaxed sources, overall. However, previous literature has also suggested that use of individual untaxed sources, such as purchases made from another person, may be more likely to occur among smokers with lower SES. In New York City, after a large tax increase, there was a rise in illegal street sales, especially in low income neighborhoods [19]. Moreover, purchases made from another person were much more common among Blacks than any other ethnic group [7,19], and were also clustered in low-income neighborhoods [19]. Therefore, further research may be needed to fully understand the differences in purchases of low/untaxed sources by socio-economic status in populations where various low and untaxed sources are readily available and use is more common.

Although use of low/untaxed sources only represents a small proportion of price minimizing strategies in this study, they represent sources of tobacco products with the lowest mean price overall, and apart from their adverse effects on tobacco-related harms, also create social harms through the illegal behavior they foster. Use of low and untaxed sources may represent a significant public health concern in the near future if it is not adequately controlled. Recently in the United States, efforts have been put in place to eliminate internet and mail order cigarettes and smokeless tobacco purchases through the Prevent All Cigarette Trafficking Act (S.1147: PACT ACT) [38]. However, other policies will be needed to curb use of other low/untaxed sources, especially those which represent a greater proportion of tax avoidance, such as duty-free, cross-border shopping, and Indian Reservations in North America.

### 4.1. Limitations

Although this study has several strengths including a large probability sample of smokers across four countries, detailed purchasing behavior information, and survey questions that have been validated for use in this international population, there are also limitations. First, all data presented are based on self reported responses, and cannot be validated by other means. Second, the data presented here are cross-sectional and we have not explored changes in such behaviors in response to price or tax increases. The ITC data can be used in this way and that form of analysis is in our future agenda.

Third, the SES measure is quite a broad one and attempts to equate across four countries with different average incomes and somewhat different educational systems. Also, the SES measure, like all others is a broad indicator of a complex construct and thus it may not accurately represent some respondents’ SES. However, we have performed many sensitivity analyses involving our composite variable to look for any differences and the resulting point estimates were essentially the same regardless of coding. Moreover, we were most interested in the low SES group, which always consisted of respondents who had both low income and low education. Thus it seems unlikely that any misattributions have substantially altered the overall patterns found.

Additionally, respondents who had missing data in either income or educational characteristics were not included in this analysis, possibly introducing bias. However, missing data was generally distributed equally between each of the price and tax avoidance behaviors studied, therefore any bias introduced would most likely be non-differential, and would bias estimates toward the null. More detailed individual data relating to a socio-economic status may be needed to fully understand the relationship of SES and use of price and tax avoidance behaviors. Further, surveys of this kind typically under-represent the very poor, as, among other reasons, many of them do not live in conventional households with land-line phones, and are thus outside the sampling frame.

There are also limitations in the range of price minimizing strategies investigated. Although we identified many of the popular price minimizing strategies, our data does not allow for identification of cigarettes which came through illicit channels which were sold through conventional channels. Measuring smuggling practices is difficult to accomplish due to its illegal nature, but this could represent a significant proportion of lower priced cigarettes which have not been adequately characterized in this study. Also, we have not been able to estimate levels of purchase of full-tax paid cigarettes sold at genuine discounts (e.g., by high turnover outlets). To do this would require estimating the base price for all jurisdictions and identifying discount purchasing as paying low relative prices for that jurisdiction, something we hope to do in future work.

Additionally, use of coupons and price promotions were not included, which may also serve to lower tobacco prices. A recent report of the International Agency for Research on Cancer (IARC) found that tobacco industry price discounting strategies do mitigate the impact of tobacco excise tax increases [39]. Additionally, the tobacco industry has very large annual expenditures on advertising and promotional discounting strategies [40], thus this price minimizing strategy should be addressed in future research.

### 4.2. Conclusions

The results support concerns that the availability of lower priced cigarette alternatives may attenuate public health efforts aimed at reducing smoking prevalence through price and tax increases because many smokers rely on them to continue their usual smoking behaviors. As individuals with lower SES may be more likely to utilize price-minimizing behaviors in general, interventions aimed at eliminating or reducing the price differentials and availability of these cheaper alternatives may be a useful tool to reduce SES differentials in smoking. However, the success of these strategies will be dependent on ensuring that illicit alternatives do not proliferate, and that strategies are put in place to minimize the adverse economic consequences on those poor smokers who are unable to quit.

## Figures and Tables

**Table 1:** Demographic Characteristics of Smokers at Wave 5, Overall (n = 7,038) and stratified by country (CA: n = 1,741; US: n = 1,790; UK: n = 1,706; AU: n = 1,801) (n, weighted %).

Characteristic	Overall (n = 7,038)	Canada (n = 1,741)	United States (n = 1,790)	United Kingdom (n = 1,706)	Australia (n = 1,801)
**Country**					
CA	1,741 (24.6)	NA	NA	NA	NA
US	1,790 (25.4)				
UK	1,706 (24.4)				
AU	1,801 (25.6)				

**Sex**					
Female	4,040 (47.6)	1,004 (46.5)	1,057 (46.2)	975 (51.5)	1,004 (46.4)
Male	2,998 (52.4)	737 (53.5)	177 (53.8)	731 (48.5)	797 (53.6)

**Age at Wave 5**					
18–39	2,120 (42.2)	521 (37.1)	456 (41.6)	488 (44.6)	655 (45.1)
40–54	2,805 (35.3)	751 (39.1)	725 (37.4)	621 (30.6)	708 (33.9)
55+	2113 (22.5)	469 (23.5)	609 (21.0)	597 (24.7)	438 (21.0)

**Ethnicity**					
White	6,290 (87.3)	1,581 (91.0)	1,487 (76.3)	1,632 (94.2)	1,599 (88.2)
Non-White	739 (12.7)	160 (9.0)	307 (23.7)	71 (5.8)	201 (11.8)

**Income**					
Low	2,207 (29.3)	471 (25.7)	636 (34.9)	578 (30.5)	522 (26.1)
Moderate	2,304 (33.1)	605 (36.0)	615 (32.5)	525 (32.3)	559 (31.4)
High	2,028 (30.8)	536 (31.1)	438 (26.8)	452 (29.2)	602 (36.0)
No Answer	499 (6.8)	129 (7.1)	101 (5.8)	151 (8.0)	118 (6.5)

**Education**					
Low	3,731 (53.2)	830 (49.0)	786 (45.8)	1,000 (56.0)	1,115 (62.0)
Moderate	2,141 (30.7)	612 (34.5)	660 (36.5)	456 (29.1)	413 (23.0)
High	1,141 (15.8)	295 (16.4)	341 (17.6)	234 (14.2)	271 (14.9)
No Answer	25 (0.2)	4 (0.1)	3 (0.1)	16 (0.7)	2 (0.1)

**SES**					
High	2,334 (33.8)	689 (39.3)	669 (36.0)	481 (31.6)	495 (28.6)
Moderate	2,767 (40.3)	624 (36.8)	673 (39.1)	653 (38.9)	817 (46.2)
Low	1,420 (18.8)	298 (16.7)	346 (19.1)	407 (20.9)	369 (18.6)
Unknown	517 (7.0)	130 (7.2)	102 (5.8)	165 (8.6)	120 (6.6)

**CPD**					
1–10	2,123 (31.0)	550 (31.0)	539 (32.0)	530 (32.1)	504 (29.0)
11–20	3,281 (46.5)	773 (43.9)	849 (46.0)	920 (54.4)	739 (41.7)
21–30	1,198 (17.0)	338 (20.6)	239 (13.9)	179 (9.7)	442 (23.5)
31+	427 (5.6)	79 (4.5)	160 (8.1)	75 (3.7)	113 (5.8)

**Minutes to 1st cig**					
>60	1,118 (16.7)	255 (14.9)	249 (16.3)	274 (16.5)	340 (19.1)
31–60	1,315 (19.9)	332 (19.8)	321 (18.4)	340 (22.4)	322 (19.0)
6–30	3,112 (43.4)	788 (44.7)	757 (41.1)	767 (43.6)	800 (44.2)
<5	1,443 (20.0)	359 (20.6)	448 (24.2)	312 (17.4)	324 (17.6)

**Smoking Status**					
Daily	6,653 (94.0)	1,646 (93.9)	1,705 (94.5)	1,624 (94.9)	1,678 (92.7)
Weekly	308 (5.0)	72 (4.7)	75 (5.0)	71 (4.7)	90 (5.6)
Monthly	77 (1.0)	23 (1.3)	10 (0.5)	11 (0.5)	33 (1.7)

**All by country comparisons are statistically significant at p < 0.05 based on the chi square test.**					

**Table 2:** Categorized List of Premium, Discount, and Roll Your Own (RYO) Brands by Country, wave 5 of the ITC-4 Survey (2006–2007).

Country	Premium Brands	Discount Brands	RYO Brands
CA	Avanti, Belmont Belvedere, Benson and Hedges, Black Cat, Camel, Cameo, Craven A, Craven M, DuMaurier, Dunhill, Export A, Gauloise, Kool, Lucky Strike, MacDonalds, Marlboro, More, Players, Premium, Rothmans, Sportsman, Supreme, Sweet Caporal, Vantage, Viscount, Winston, Golden Leaf	Baileys, Peter Jackson, Number 7, Legend, Canadian Classic, JPS, Mark Ten, Matinee, Medallion, Podium, Smoking, Maximum, DK’s, Putters, Trad A, All Natural Natives, Discount, Sago, Mohawk, Reserve Rockets, Rollies, Saratoga, Stykes, DH, DailyMail, Canadian, Generic Cigarettes Purchased from Indian Reservation	Belvedere, Cameo, Export A, Matinee, Number 7, Players, Drum, Canadian Classic, Peter Jackson, Honey Time, Captain Black Bleu, Craven A, Extorta
US	Belair, Benson & Hedges, Camel, Capri, Carlton, Djarum, Eve, Kent, Kool, Lark, Lucky Strike, Marlboro, Merit, More, Nat Sherman, Newport, Pall Mall, Parliament, Rothmans, Salem, Tareyton, True, Vantage, Virginia Slims, Winston, American Spirit	305’s, Always Save, Natural American Spirit, Austin, Baileys, Basic, Best Value, Bridgeport, Bronco, Bronson, Carnival, Checker, Cherokee, Cheyenne, Class A, Complete, Doral, Drum, Du Maurier, Eagle, Echo, Exact, First Ones, First Choice, 1st Class, GT Ones, Generic, Golden Beach, Gold Coast, GPC, GP’s, Grand, GrandsUSA, Grand Prix, Hats Off, Highway, Hi-val, Jacks, Kings, Kingston, Kingsley, Kentucky’s Best, Liberty, Liggett, Main Street, Malibu, Market, Maxim, Mild 7, Marathon, Maverick, Misty, Monarch, Montclair, Mustang, Niagara, Native, Old Gold, OK Poker, Patriots, Poker, Pyramid, Raleigh, Roger, Sandia, Santa Fe, Seneca, Shield, Sky Dancer, Smoker Friendly, Smokers Choice, Smokin Joes, Sonoma, Sundance, Tahoe, Tourney, Tucson, USA, USA Gold, Viceroy, Wave, Westport, Winchester, Wild Horse, Yours	Bugler, Camel, Drum, Gambler, Golden Harvest, Kite, McClintock, Midnight Special, Roll Rich, Stokers, Top, Zig Zag
UK	Benson & Hedges, Berkley, Camel, Consulate, Capstan, Club, Drum, Dunhill, Davidoff, Marlboro, More, Park Drive Plain, Piccadilly, Regal, Rothmans, Silk Cut, Superkings, Lucky Strike, Raffles, Peter Stuyvesant, Players, Woodbine, American Spirit, Imperial	Dorchester, Embassy, JPS, Lambert & Butler, Mayfair, Richmond, Senior Service, Sovereign, Sterling, Balmoral, Basic, Craven A, Redband, Windsor, Gold Coast, Select, Fortuna, Goldmark, John Players, L&M, Royals	Blue Ridge, Golden Virginia, Old Holborn, Turner, Domingo, Hamlet, Bayside Gold, Amber Leaf, Cutters Choice, Drum, Gauloises, Samson, Silver Strand, St. Bruno, Café Crème, Players, Gold Leaf
AU	Alpine, Benson & Hedges, Camel, Dunhill, Escort, Kool, Marlboro, More, Peter Jackson, Peter Stuyvesant, Rothmans, Vogue, Winfield, Kent, Superkings	Brandon, Cambridge, Choice, Holiday, Longbeach, Double Happiness, Gudang Garam, Stradbroke, Horizon, Raison, Super Lights	Capstan, Champion, Dr. Pat Virginia, Drum, Marlboro, Port Royal, Stockmans, White Ox, Winfield, Old Holborn, Bank Aromatic, Havelock, Longbeach

**Table 3:** Frequency of using low and untaxed sources at wave 5 by country (weighted percentages).

	Weighted Percentage of Reported Use				

Purchasing Behavior	All	CA	US	UK	AU
**Purchase from Low/Untaxed Sources**					
No	91.7	89.9	94.6	83.2	98.7
Yes	8.3	10.1	5.4	16.8	1.3

**Specific Low/Untaxed Sources:**					
Military Commissary, Duty-free *	1.9	0.7	0.8	5.8	0.5
Indian Reservation	2.7	7.9	3.0	0.0	0.0
Outside State/Country	1.9	0.1	0.3	7.2	0.2
By Internet or Telephone *	0.2	0.0	0.5	0.3	0.0
Friend, Relative, Someone else *	1.6	1.3	0.8	3.6	0.7

**Use Discount Brand Cigarettes**					
Premium	50.7	49.0	69.5	32.7	50.6
Discount	35.9	43.5	27.8	38.3	34.4
RYO	13.5	7.5	2.6	29.0	15.0

**Purchase from Cartons**					
No	71.3	71.0	58.8	75.1	82.8
Yes	28.7	29.0	41.2	24.9	17.2

**Any Price or Tax Avoidance** **					
No	37.2	36.9	45.2	23.1	42.9
Yes	62.8	63.1	54.8	76.9	57.1

* Represents categories that have been combined from the original survey due to low responses.

** Any price or tax avoidance is a composite measure of purchasing from low/untaxed sources, using discount/generic or RYO tobacco, and purchasing cigarettes in a carton at last purchase. Respondents who reported using at least one of these behaviors at last purchase were coded as participating in any price or tax avoidance.

**Table 4:** Predictors of using Low and Untaxed Sources, Cheaper Brands/RYO, Cartons, and Any Price/Tax Avoidance Behaviors at last purchase.

		Low/untaxed (n = 6,543)		discount/RYO (n = 6,400)		cartons (n = 5,669)		“Any Use” (n = 6,374)

Predictor	N	OR (95% CI)	N	OR (95% CI)	N	OR (95% CI)	N	OR (95% CI)
**SES**								
**High**	2,346	1.00	2,307	1.00	2101	1.00	2301	1.00
**Moderate**	2,780	0.99 (0.81–1.21)	2,719	**1.36 (1.21–1.53)**	2414	0.89 (0.78–1.03)	2712	**1.15 (1.02–1.30)**
**Low**	1,417	**0.74 (0.57–0.96)**	1,374	**1.85 (1.59–2.15)**	1154	**0.57 (0.47–0.68)**	1361	**1.25 (1.07–1.45)**

**Country**								
**CA**	1,620	1.00	1,542	1.00	1472	1.00	1534	1.00
**US**	1,698	**0.58 (0.44–0.75)**	1,666	**0.41 (0.35–0.48)**	1650	**2.01 (1.71–2.37)**	1665	**0.73 (0.63–0.85)**
**UK**	1,540	**1.92 (1.55–2.38)**	1,520	**1.97 (1.69–2.28)**	1127	0.88 (0.73–1.06)	1508	**2.03 (1.73–2.39)**
**AU**	1,685	**0.13 (0.08–0.20)**	1,672	0.93 (0.80–1.07)	1420	**0.47 (0.39–0.57)**	1667	**0.76 (0.66–0.88)**

**Age**								
**18–39**	2,006	1.00	1,978	1.00	1765	1.00	1972	1.00
**40–54**	2,635	**1.65 (1.32–2.05)**	2,589	**1.32 (1.17–1.49)**	2261	**1.66 (1.43–1.92)**	2576	**1.52 (1.35–1.71)**
**55+**	1,902	**2.24 (1.77–2.83)**	1,833	**1.42 (1.24–1.64)**	1643	**3.42 (2.90–4.03)**	1826	**2.39 (2.05–2.78)**

**Sex**								
**Female**	3,740	1.00	3,658	1.00	3380	1.00	3651	1.00
**Male**	2,803	0.96 (0.80–1.14)	2,742	**0.75 (0.68–0.84)**	2289	**0.69 (0.61–0.78)**	2723	**0.69 (0.62–0.77)**

**Minority**								
**No**	5,858	1.00	5,727	1.00	5038	1.00	5703	1.00
**Yes**	685	0.91 (0.65–1.26)	673	**0.73 (0.62–0.86)**	631	**0.69 (0.56–0.84)**	671	**0.68 (0.58–0.80)**

**CPD**								
**1–10**	1,981	1.00	1,932	1.00	1744	1.00	1924	1.00
**11–20**	3,069	**1.30 (1.04–1.62)**	3,011	**1.41 (1.25–1.59)**	2641	**2.20 (1.88–2.58)**	3002	**1.75 (1.55–1.98)**
**21–30**	1,103	**1.64 (1.24–2.17)**	1,077	**1.40 (1.19–1.65)**	953	**3.50 (2.87–4.26)**	1071	**2.04 (1.72–2.04)**
**31+**	390	1.20 (0.77–1.87)	380	**2.22 (1.73–2.84)**	331	**3.36 (2.54–4.45)**	377	**2.93 (2.23–3.84)**

All **Bolded** values represent significant associations at p < 0.05 level.

All variables were entered into a multivariate logistic regression model to assess the likelihood of using each price/tax avoidance behavior among current smokers included in the wave 5 (main or replenishment) survey.

In Overall Model: p value for interaction between SES and other covariates are as follows:

***Low/Untaxed***: country: 0.125, age: 0.356, sex: 0.612, minority: 0.367, CPD: 0.525; ***Discount/RYO***: country: 0.301, age: 0.054, sex: 0.337, **minority: 0.027** *, CPD: 0.432;

***Cartons***: country: 0.182, age: 0.668, sex: 0.098, minority: 0.110, CPD: 0.252; ***Any use***: country: 0.075, age: 0.086, sex: 0.196, minority: 0.016 *, CPD: 0.224.

**Table 5:** Price per cigarette overall and by country for various price and tax avoidance techniques at last purchase (excluding RYO users *) and for a sub-sample that excludes UK respondents.

	Total Sample (n = 5,939) mean (SD)	Excluding UK (n = 4,744) ** mean (SD)	CA (n = 1,533) mean (SD)	US (n = 1,726) mean (SD)	UK (n = 1,195) mean (SD)	AU (n = 1,485) mean (SD)
**Country**						
**CA**	0.28 (0.082)					
**US**	0.18 (0.068)					
**UK**	0.43 (0.105)					
**AU**	0.31 (0.044)					

**Low/Untaxed**						
**Full**	0.30 (0.113)	0.26 (0.084)	0.30 (0.063)	0.18 (0.067)	0.46 (0.065)	0.31 (0.041)
**Low/No**	0.16 (0.085)	0.13 (0.069)	0.12 (0.069)	0.13 (0.052)	0.21 (0.089)	0.19 (0.113)

**Discount Use**						
**Premium Brand**	0.29 (0.113)	0.27 (0.086)	0.32 (0.064)	0.19 (0.059)	0.44 (0.124)	0.33 (0.043)
**Discount Brand**	0.28 (0.117)	0.23 (0.082)	0.25 (0.071)	0.14 (0.059)	0.42 (0.081)	0.28 (0.031)

**Carton Use**						
**No**	0.32 (0.105)	0.28 (0.075)	0.31 (0.066)	0.20 (0.065)	0.46 (0.058)	0.31 (0.040)
**Yes**	0.21 (0.110)	0.19 (0.087)	0.23 (0.092)	0.14 (0.049)	0.32 (0.143)	0.28 (0.054)

**Any Use**						
**None**	0.32 (0.104)	0.29 (0.076)	0.34 (0.050)	0.21 (0.056)	0.50 (0.052)	0.33 (0.034)
**Any (1 + Sources)**	0.27 (0.117)	0.22 (0.083)	0.26 (0.072)	0.15 (0.054)	0.40 (0.107)	0.28 (0.039)

Currency adjusted to $USD using the website: OANDA, found at http://www.oanda.com/convert/classic (accessed 27 July 2010).

Date used for currency conversions was December 14th 2006 as it was the midpoint between the beginning and end of data collection for wave 5 (11 October 2006 to 17 February 2007). Adjustment rates are as follows: 1 CAD = 0.86754 USD; 1 GBP = 1.96915 USD; 1 AUD = 0.78737 USD.

* RYO Users excluded—price per “cigarette” cannot be calculated.

** Average price per cigarette for the sample is also given excluding UK participants (n = 4744). In the UK, prices overall are higher, but in particular discount brand prices are high. Due to the high percentage of discount cigarette users in the UK, the UK prices were disproportionately weighing the overall average, resulting in a smaller price differential between discount and premium brands.
